# Tobacco and E-cigarette use among cancer survivors in the United States

**DOI:** 10.1371/journal.pone.0226110

**Published:** 2019-12-09

**Authors:** Ramzi G. Salloum, Jinhai Huo, Ji-Hyun Lee, Juhan Lee, Jesse Dallery, Thomas George, Graham Warren

**Affiliations:** 1 Department of Health Outcomes and Biomedical Informatics, College of Medicine, University of Florida, Gainesville, Florida, United States of America; 2 Division of Quantitative Sciences, University of Florida Health Cancer Center, Department of Biostatistics, University of Florida, Gainesville, Florida, United States of America; 3 Department of Health Education and Behavior, University of Florida, Gainesville, Florida, United States of America; 4 Department of Psychology, University of Florida, Gainesville, Florida, United States of America; 5 Division of Hematology/Oncology, Department of Medicine, College of Medicine, University of Florida, Gainesville, Florida, United States of America; 6 Department of Radiation Oncology, Medical University of South Carolina, Charleston, South Carolina, United States of America; 7 Department of Cell and Molecular Pharmacology, Medical University of South Carolina, Charleston, South Carolina, United States of America; University of Calfornia San Francisco, UNITED STATES

## Abstract

**Background:**

Limited information exist on tobacco and e-cigarette use patterns in cancer survivors. The purpose of this study is to report on use patterns in cancer survivors compared with non-cancer participants from the Population Assessment of Tobacco and Health (PATH) Study.

**Methods:**

Sociodemographic data and tobacco product use were analyzed for 32,244 adult participants from the PATH Study in 2013–2014 by cancer status and age. Logistic regression examined the patterns of and factors associated with tobacco use by cancer status.

**Results:**

Overall, cancer survivors represented 7.1% (n = 1,527) of participants, were older, and had a higher proportion of females and non-Hispanic whites than non-cancer participants. In cancer survivors, current and former cigarette smoking was reported in 12.7% and 32.9% respectively, compared with 18.5% and 19.0% in non-cancer adults. Current e-cigarette use was reported by 3.8% of survivors compared with 5.7% of non-cancer participants. Dual tobacco use was reported by 25.0% and poly use by 6.9% of cancer survivors who currently smoked. All other forms of current tobacco use were individually reported by <5% of survivors. Young adult cancer survivors (aged 18–44) reported the highest rates of current cigarette smoking (27.9%) and current e-cigarette use (11.8%). The effects of age, sex, race/ethnicity, education, and income on tobacco use status were comparable for cancer survivors and non-cancer participants. Cancer survivors who were younger, male, of lower educational attainment, and those diagnosed with a tobacco-related cancer were more likely to report current tobacco use.

**Conclusions:**

Among cancer survivors, cigarette smoking remains the predominant form of tobacco use, although other tobacco/nicotine use and dual/poly use are common. The PATH Study provides detailed tobacco product use patterns in survivors, including their adoption of emerging alternative tobacco products.

## Introduction

Many cancer survivors continue to use tobacco products after their cancer diagnosis despite the mounting evidence showing reduced effectiveness of cancer treatments, increased overall and cancer-specific mortality and increased risk for a second primary cancer.[1] Tobacco use has also been associated with poorer response to cancer treatment and cancer recurrence leading to significantly increased costs associated with cancer treatment.[2] Cigarette smoking rates among cancer survivors have been reported in prior studies using two national data sources. First, using the National Health Interview Survey (NHIS), current smoking prevalence among cancer survivors was 20.2% in a study that combined four waves of the NHIS (1998–2001).[3] Second, using the National Cancer Institute (NCI)’s Health Information National Trends Survey (HINTS), current smoking prevalence was 18.7% in a combined three waves of HINTS (2003, 2005, and 2007).[4]

The use of e-cigarettes and other tobacco products have increased considerably in recent years,[5,6] and a recent analysis of the NHIS showed an increasing trend in the prevalence of e-cigarette ever use among cancer survivors from 8.5% in 2014–2015 to 10.7% in 2016–2017.[7] Another analysis of the 2014–2015 NHIS focusing on the use of e-cigarettes among adults with medical comorbidities found that former smokers with cancer had lower odds of e-cigarette use.[8] Further, a recent study of cancer patients at Mid-South cancer centers found that among cigarette smokers, nearly one-third reported using two or more tobacco products.[9]

The Population Assessment of Tobacco and Health (PATH) Study was established in 2011 following the Family and Smoking Prevention and Tobacco Control Act of 2009.[10] The purpose of the PATH Study is to produce national epidemiologic information on tobacco use behavior and health in the U.S. population.[11] In contrast to other national surveys that have been used for the surveillance of tobacco use, the PATH Study provides a detailed assessment of use behaviors for the various nicotine and tobacco products available in the U.S. in a manner that is in more depth than prior population health assessments.[11,12]

A recent analysis of data from the PATH Study examined dual use of cigarettes and e-cigarettes in cancer survivors and found that among smokers, cancer survivors were using e-cigarettes at similar rates as non-cancer participants and both groups were motivated to use e-cigarettes largely for perceived health-related reasons.[12] Given its focus on dual use, the analysis excluded participants who were not current cigarette smokers and it did not investigate the use of other tobacco products. Therefore, the current study examines tobacco and e-cigarette use information in cancer survivors who participated in the PATH Study to address a knowledge gap in characterizing use patterns for cigarettes and alternative products from a nationally representative sample of U.S. adults.

## Methods

### Data source

Data were obtained from the PATH Study, a household-based, longitudinal, nationally representative cohort study of 45,971 adults and youth in the U.S. that is designed to measure prevalence and correlates of tobacco use. The current study was limited to adults who participated in Wave 1 of the PATH Study between September 2013 and December 2014. PATH recruitment was completed using a four-stage, stratified probability sample design in which a predetermined number of participants (N = 32,320 for adults) was randomly recruited by home address. The sample included current, former, and never tobacco users who completed computer- and audio-assisted structured interviews and received $35 compensation. The time required to complete the survey was approximately 45 minutes. The PATH Study was weighted to reflect the U.S. population including adjustments for oversampling and nonresponse.[13] The weighted interview response rate for adults in Wave 1 was 74.0%. Additional details of survey methodology are available elsewhere.[11] Although data from subsequent waves of the PATH Study are available, we limited the analysis to Wave 1 due to missing capture of the former tobacco use status measures in Waves 2 and 3, which impacts the ability to estimate reliable prevalence rates.

### Tobacco and nicotine use categories

The PATH Study uses pictures to assist respondents in answering questions about their awareness and use of noncigarette tobacco and nicotine products. Prevalence of current, former, or never use was assessed for the following tobacco and nicotine products: cigarettes, e-cigarettes (electronic cigarettes), traditional cigars, cigarillos, filtered cigars, smokeless tobacco, snus, pipe tobacco, and hookah. For cigarettes, current smoking status was assigned to participants who had smoked more than 100 cigarettes in their lifetime and currently smoke cigarettes every day or some days, and former smoking was assigned to those who had smoked at least 100 cigarettes in their lifetime but now do not smoke at all. Never smoking was assigned to those who had never smoked a cigarette, even one or two puffs. Current cigarette smokers were further classified into daily and less than daily smokers. Pack-year history was also calculated for current and former cigarette smokers. For all other tobacco and nicotine-delivery products, current use was assigned to those who have ever used the product and now use it every day or some days, whereas former use was assigned to those who had ever used the product fairly regularly but now do not use it at all. Never use was assigned to those who had never used the product even once or twice. Among current users of any tobacco/nicotine products, we further classified product use into mono use (1 product only), dual use (any 2 products), or poly use (>2 products).

### Reasons for noncigarette product use

Participants who reported current use of any noncigarette product or who had quit such products in the past 12 months were asked a series of yes or no questions about 13 reasons for using these products. There were 3 health-related reasons—“they might be less harmful to me than cigarettes,” “they might be less harmful to people around me than cigarettes,” and “using them helps people to quit smoking cigarettes.” The remaining reasons were not health-related, including, “I can use them at times when or in places where smoking cigarettes isn’t allowed,” “they don’t smell,” “they are more acceptable to non-tobacco users,” “they come in flavors I like.” Each reason was analyzed separately.

### Cancer status

Participants were defined as cancer survivors if they responded affirmatively to the following question, ‘Have you ever been told by a doctor or other health professional that you had cancer?’ Cancer survivors were further classified as having a tobacco-related cancer if the cancer site reported was one of the following: bladder, cervix, colon, esophagus, kidney, larynx, liver, lung, mouth, pancreas, rectum, stomach, and throat.

### Sociodemographic measures

The following demographic characteristics of participants were included: gender (male or female), age (in years) aggregated into groups (18–44, 45–64, and 65 or more), race/ethnicity (non-Hispanic white, non-Hispanic black, and other), educational attainment (not a high school graduate, GED or high school graduate, some college or associate degree, bachelor’s degree or higher), annual household income (less than $25,000, $25,000 to $49,999, $50,000 to $99,999, and $100,000 or more), and U.S. Census region (Northeast, South, Midwest, and West).

### Statistical analyses

Frequencies and percentages weighted to the U.S. population were calculated by cancer status (i.e., cancer survivor vs. no history of cancer) and age group across all adults who completed the survey for current/former/never use of the following nicotine and tobacco product categories: cigarettes, e-cigarettes, any cigars (i.e., cigars, cigarillos, and filtered cigars), smokeless tobacco and snus, pipe tobacco, and hookah. We also classified participants who had used any tobacco/nicotine products as mono (one product), dual (two products), and poly (more than two) tobacco/nicotine product users among those who reported current use of any types of tobacco or nicotine products. Weighted percentages were calculated for use of the aforementioned categories by cigarette smoking status and stratified by cancer status (cancer survivor vs. no history of cancer). Among cancer survivors, weighted percentages and 95% confidence intervals (CIs) were calculated using the logit transformation method[5] to describe the leading reasons for noncigarette product use. The differences in demographic characteristics by cancer status were assessed using Rao-Scott adjusted Pearson Chi-square tests. Differences in continuous variables (e.g., pack-year history) by cancer status were assessed using adjusted Wald tests and p-values were estimated based on F-distributions. Multivariable logistic regression was used to identify demographic and socioeconomic characteristics associated with (1) any tobacco or nicotine product use and (2) dual/poly use of at least two products. The models reported estimated adjusted odds ratios (AORs) and their 95% CIs for each independent variable. The models for cancer survivors included as an additional variable whether a tobacco-related cancer was reported. Percentages and AOR estimates were weighted to the U.S. adult population to account for the complex sampling scheme using PATH Survey weights, and 95% CIs were estimated with the method of balanced repeated replications[14] with Fay’s adjustment set to 0.3 to increase estimate stability.[15] We excluded cases with missing cancer status. All statistical analyses were conducted using STATA/SE software (version 15.1; StataCorp, College Station, TX, USA) survey procedures using the “svy:” command, and all statistical tests were two-tailed. All analyses were prepared in a reproducible manner and available upon request. This study was deemed exempt by the University of Florida Institutional Review Board.

## Results

### Sample characteristics

Characteristics of adult respondents in Wave 1 of the PATH Study, stratified by cancer status, are presented in Table 1. Cancer survivors (n = 1,527) represented 7.1% of the overall sample (weighted prevalence), and were generally older than non-cancer respondents: 10.6% of cancer survivors were under age 45 as compared with 50.1% of non-cancer respondents. Among cancer survivors, 58.2% were female, compared with 51.4% among non-cancer respondents. Only 10.6% of cancer survivors were from racial or ethnic minority groups, compared with 23.0% of non-cancer respondents. Though statistically different, patterns of education and household income were similar in cancer survivors and non-cancer participants. Almost one-third of cancer survivors (28.7%) reported having been diagnosed with a tobacco-related cancer.

**Table 1:** Baseline characteristics of adult respondents (*N* = 32,320) by cancer status: Population Assessment of Health and Tobacco (PATH) Study, 2013–2014.

Characteristics	Cancer survivor (n = 1,527)		No history of cancer (n = 30,717)		
	n	%	n	%	*P* value
**Age, in years**					<0.001
18–44	296	10.6	20,054	50.1	
45–64	653	38.0	8,141	34.3	
≥65	578	51.4	2,516	15.6	
**Sex**					<0.001
Female	898	58.3	15,061	51.4	
Male	629	41.7	15,656	48.6	
**Race/ethnicity**					
White, non-Hispanic	1,298	89.5	22,531	77.0	<0.001
Black, non-Hispanic	133	6.0	4,904	12.8	
Other	96	4.6	3,282	10.2	
**Education**					
Not high school graduate	186	10.1	4,039	11.7	0.051
GED or high school graduate	417	29.1	9,333	29.5	
Some college or associate degree	511	29.5	10,780	31.2	
Bachelor’s degree or higher	408	31.3	6,393	27.6	
**Annual household income**					
Less than $25,000	669	38.2	14,859	41.5	0.207
$25,000 to $49,999	314	21.2	6,352	20.5	
$50,000 to $99,999	315	23.5	5,821	22.0	
$100,000 or more	229	17.2	3,685	16.0	
**U.S. Census region**					
Northeast	226	16.7	4,811	18.3	0.002
Midwest	426	25.8	7,251	21.1	
South	535	36.4	11,658	37.2	
West	340	21.2	6,997	23.4	
**Tobacco-related cancer**					
Yes	538	28.7	NA	NA	NA
No	979	71.4	NA	NA	

Note: Tobacco related cancers include bladder, cervix, colon, esophagus, kidney, larynx, liver, lung, mouth, pancreas, rectum, stomach, and throat cancer. 76 cases were excluded because cancer status was missing.

Abbreviation: NA, not applicable

### Tobacco and nicotine product use

Weighted prevalence of current, former, and never use of tobacco and nicotine products stratified by cancer status is reported in Table 2. Overall, 17.2% of cancer survivors were current users of any tobacco or nicotine product (12.6% were daily users), compared with 26.9% (18.1% daily users) among non-cancer respondents. The most common currently used product was conventional cigarettes among both cancer survivors (12.7%; 10.5% were daily smokers) and non-cancer respondents (18.5%; 14.7% were daily smokers), followed by any cigars (4.2 vs. 7.6%), e-cigarettes (3.8% vs. 5.7%), and smokeless tobacco and snus (1.6% vs. 3.4%), hookah (1.0% vs. 4.3%), and pipe tobacco (0.9% vs. 1.1%). Among cancer survivors on average, current smokers had a 28.9 pack-year history compared to a 19.6 pack-year history among non-cancer respondents.

**Table 2:** Tobacco and nicotine product use stratified by cancer status: Population Assessment of Health and Tobacco (PATH) Study, 2013–2014.

Tobacco/nicotine product	Cancer survivor (n = 1,527)	No history of cancer (n = 30,717)	
	Weighted % (95% CI)	Weighted % (95% CI)	*P* value
**Cigarettes**			
Current smoker	12.7 (11.3, 14.1)	18.5 (18.0, 19.0)	<0.001
Pack-years, mean (SE)	28.9 (1.1)	19.6 (0.50)	<0.001
Daily smoker	10.5 (9.3, 12.0)	14.7 (14.2, 15.2)	
Less than daily smoker	2.1 (1.6, 2.7)	3.8 (3.6, 4.0)	
Former smoker	32.9 (29.9, 36.0)	19.0 (18.2, 19.9)	
Pack-years, mean (SE)	29.0 (2.6)	21.2 (1.05)	0.005
Never smoker	54.5 (51.0, 57.9)	62.5 (61.4, 63.6)	
**E-cigarettes**			
Current user	3.8 (3.1, 4.7)	5.7 (5.4, 5.9)	<0.001
Daily user	1.0 (0.7, 1.4)	1.2 (1.1, 1.3)	
Less than daily user	2.8 (2.2, 3.6)	4.5 (4.3, 4.7)	
Former user	0.7 (0.4, 1.0)	1.0 (1.0, 1.1)	
Never user	95.5 (94.6, 96.3)	93.3 (93.0, 93.6)	
**Any cigars (cigars, cigarillos, and filtered cigars)**			
Current smoker	4.2 (3.5, 5.1)	7.6 (7.3, 7.9)	<0.001
Daily smoker	0.8 (0.5, 1.3)	0.9 (0.8, 1.0)	
Less than daily smoker	3.4 (2.7, 4.2)	6.7 (6.5, 7.0)	
Former smoker	4.2 (3.3, 5.5)	3.3 (3.0, 3.6)	
Never smoker	91.6 (90.1, 92.8)	89.1 (88.7, 89.5)	
**Smokeless tobacco and snus**			
Current user	1.6 (1.2, 2.1)	3.4 (3.2, 3.7)	<0.001
Daily user	0.7 (0.5, 1.0)	1.8 (1.6, 2.0)	
Less than daily user	0.9 (0.6, 1.4)	1.6 (1.5, 1.8)	
Former user	2.5 (1.8, 3.4)	3.3 (3.0, 3.6)	
Never user	95.9 (94.9, 96.8)	93.3 (92.8, 93.7)	
**Hookah**			
Current smoker	1.0 (0.7, 1.4)	4.3 (4.0, 4.6)	<0.001
Daily smoker	NA	0.1 (0.1, 0.1)	
Less than daily smoker	0.9 (0.6, 1.4)	4.2 (3.9, 4.5)	
Former smoker	0.2 (0.1, 0.4)	0.8 (0.7, 0.9)	
Never smoker	98.9 (98.4, 99.2)	94.9 (94.6, 95.2)	
**Pipe tobacco**			
Current smoker	0.9 (0.6, 1.2)	1.1 (1.0, 1.3)	<0.001
Daily smoker	0.2 (0.1, 0.4)	0.1 (0.1, 0.1)	
Less than daily smoker	0.6 (0.4, 1.0)	1.0 (0.9, 1.2)	
Former smoker	6.6 (5.2, 8.4)	2.3 (2.1, 2.6)	
Never smoker	92.5 (90.7, 94.0)	96.6 (96.3, 96.8)	
**Any product**			
Current user	17.2 (15.5, 19.0)	26.9 (26.3, 27.5)	<0.001
Daily user	12.6 (11.2, 14.2)	18.1 (17.6, 18.7)	
Less than daily user	4.6 (4.0, 5.3)	8.8 (8.5, 9.1)	
Former user	33.1 (30.0, 36.3)	18.2 (17.4, 19.1)	
Never user	49.7 (46.2, 53.3)	54.9 (53.7, 56.0)	

Notes: Data were collected from September 12, 2013, through December 15, 2014. The columns in the table are not mutually exclusive; participants who used one product may also have used another product. Percentages were weighted to the U.S. population and confidence intervals were estimated with the method of balanced, repeated replications. Complete data about every type of tobacco product were required to define nonuse of any tobacco; similarly, complete data about every type of cigar were required to define nonuse of any cigar, and complete data about smokeless tobacco and snus were required to define nonuse of smokeless tobacco including snus pouches. P-values for pack-year history are the result of the adjusted Wald test and estimated based on F distribution.

Abbreviation: NA, not available (suppressed)

Former use of any tobacco or nicotine product was higher in cancer survivors as compared with non-cancer participants (33.1% vs. 18.2%) as was former use of conventional cigarettes (32.9% vs. 19.0%), pipe tobacco (6.6% vs. 2.3%), and any cigars (4.2% vs. 3.3%). In contrast, lower prevalence of former tobacco use was reported among cancer survivors vs. non-cancer respondents for e-cigarettes (0.7% vs. 1.0%), smokeless tobacco and snus (2.5% vs. 3.3%) and hookah (0.2% vs. 0.8%). Among cancer survivors on average, former smokers had a 29.0 pack-year history compared to 21.2 pack-year history among non-cancer respondents.

**Fig 1:**
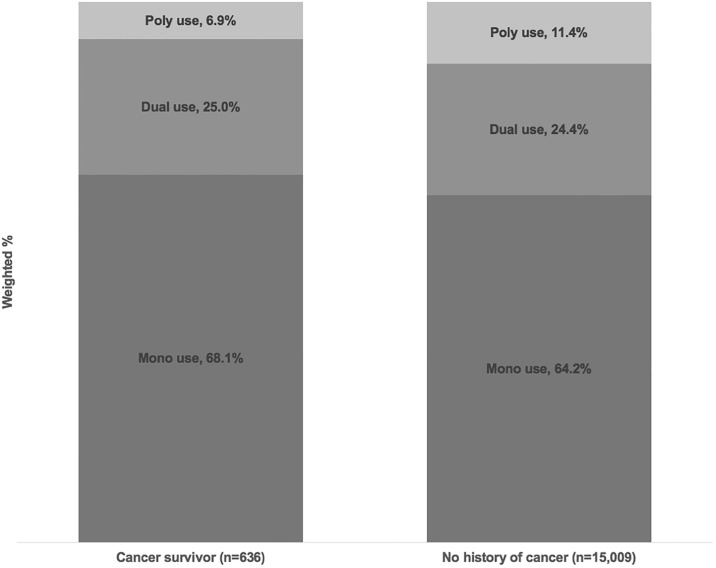
Mono, dual and poly tobacco/nicotine use among current users of any tobacco/nicotine products, by cancer status. Note: 44 cancer survivors and 766 adults with no history of cancer were excluded due to missing values for any tobacco or nicotine product use.

Among cancer survivors who were current tobacco or nicotine product users and reported complete information on product use (n = 636), 68.1% used only one product (mono users), 25.0% were dual users, and 6.9% used more than two products (Fig 1). Among non-cancer respondents (n = 15,009), 64.2% were mono users, 24.4% dual users, and 11.4% poly users.

Table 3 shows the weighted prevalence of current, former, and never use of tobacco and nicotine products among cancer survivor stratified by age group (i.e., 18–44, 45–64, and 65 or more). Current cigarette smoking was highest among the 18–44 age group (27.9%) and former smoking was highest among those 65 or more (40.9%). Young adult cancer survivors (18–44 years) had the highest rates any tobacco use (37.7%) across all age groups, as well as for the following individual products: e-cigarette use (11.8%), cigar smoking (10.6%), hookah smoking (6.2%), and smokeless tobacco/snus (3.7%).

**Table 3:** Tobacco and nicotine product use among cancer survivors, stratified by age group: Population Assessment of Health and Tobacco (PATH) Study, 2013–2014.

Cancer survivor Age group, in years	18–44 (n = 296)	45–64 (n = 653)	≥65 (n = 578)	
Tobacco/nicotine product	Weighted % (95% CI)	Weighted % (95% CI)	Weighted % (95% CI)	*P* value
**Cigarettes**				<0.001
Current smoker	27.9 (22.5, 34.0)	16.9 (14.4, 19.8)	6.4 (5.2, 7.8)	
Daily smoker	23.1 (18.3, 28.8)	13.5 (11.3, 16.1)	5.8 (4.7, 7.1)	
Less than daily	4.7 (2.9, 7.7)	3.4 (2.5, 4.6)	0.6 (0.3, 1.1)	
Former smoker	17.6 (12.6, 24.1)	26.3 (22.4, 30.6)	40.9 (35.8, 46.2)	
Never smoker	54.5 (47.1, 61.7)	56.8 (52.0, 61.4)	52.7 (47.3, 58.1)	
**E-cigarettes**				<0.001
Current user	11.8 (8.6, 16.0)	5.4 (4.0, 7.2)	1.0 (0.6, 1.7)	
Daily user	2.7 (1.5, 4.6)	1.5 (0.9, 2.5)	0.3 (0.1, 0.6)	
Less than daily	9.2 (6.4, 12.9)	3.9 (2.7, 5.4)	0.8 (0.4, 1.4)	
Former user	1.8 (0.9, 3.8)	0.9 (0.5, 1.7)	0.3 (0.1, 0.8)	
Never user	86.4 (81.9, 89.8)	93.8 (91.8, 95.3)	98.7 (97.9, 99.2)	
**Any cigars**				<0.001
Current smoker	10.6 (7.6, 14.6)	5.2 (3.9, 6.9)	2.1 (1.5, 3.1)	
Daily smoker	NA	1.0 (0.6, 1.7)	0.7 (0.3, 1.5)	
Less than daily	9.7 (6.7, 13.7)	4.2 (3.1, 5.8)	1.4 (0.9, 2.2)	
Former smoker	3.6 (2.0, 6.5)	3.3 (2.1, 5.2)	5.1 (3.4, 7.4)	
Never smoker	85.8 (81.3, 89.3)	91.5 (89.1, 93.4)	92.8 (90.5, 94.6)	
**Smokeless tobacco and snus**				0.023
Current user	3.7 (2.0, 6.7)	1.9 (1.3, 2.9)	0.9 (0.5, 1.5)	
Daily user	NA	1.0 (0.5, 1.7)	0.5 (0.2, 0.9)	
Less than daily	2.7 (1.3, 5.5)	1.0 (0.5, 1.8)	NA	
Former user	4.3 (1.7, 10.4)	2.7 (1.6, 4.3)	1.9 (1.2, 3.3)	
Never user	92.0 (86.2, 95.5)	95.4 (93.6, 96.7)	97.2 (95.8, 98.1)	
**Hookah**				<0.001
Current smoker	6.2 (4.1, 9.5)	NA	NA	
Daily smoker	NA	NA	NA	
Less than daily	6.2 (4.1, 9.5)	NA	NA	
Former smoker	NA	NA	NA	
Never smoker	92.9 (89.5, 95.3)	99.3 (98.4, 99.7)	99.8 (99.5, 100.0)	
**Pipe tobacco**				<0.001
Current smoker	NA	1.0 (0.7, 1.7)	NA	
Daily smoker	NA	NA	NA	
Less than daily	NA	0.8 (0.4, 1.4)	NA	
Former smoker	NA	3.7 (2.4, 5.7)	9.9 (7.3, 13.2)	
Never smoker	96.7 (94.0, 98.3)	95.3 (93.4, 96.6)	89.6 (86.4, 92.1)	
**Any product**				<0.001
Current user	37.7 (31.4, 44.4)	22.5 (19.4, 26.0)	9.1 (7.6, 10.8)	
Daily user	26.9 (21.6, 32.9)	16.3 (13.8, 19.2)	6.9 (5.7, 8.5)	
Less than daily	10.8 (7.8, 14.8)	6.2 (4.8, 7.9)	2.1 (1.5, 3.0)	
Former user	15.9 (10.8, 22.7)	25.1 (21.2, 29.4)	42.6 (37.4, 47.9)	
Never user	46.5 (38.6, 54.5)	52.5 (47.6, 57.3)	48.4 (43.1, 53.7)	

Rao-Scott adjusted Pearson Chi-square tests were performed to test statistical significant associations between tobacco/nicotine use status and age group.

Abbreviation: NA, not available (suppressed)

### Dual and poly use of cigarettes and non-cigarette products

Table 4 displays the weighted prevalence of current, former, and never use of tobacco and nicotine products evaluated against current, former, and never cigarette smoking status by cancer status. Among cancer survivors who reported current cigarette smoking, 58.8% were exclusive cigarette smokers, whereas 22.0% also reported current use of e-cigarettes, 15.0% were current any cigar smokers, 4.7% were also current users of smokeless tobacco or snus, 4.0% were also current hookah smokers, and 2.0% were also current smokers of pipe tobacco. On average, cancer survivors who were exclusive cigarette smokers reported smoking 16.7 cigarettes per day, compared with 15.8 cigarettes per day among current e-cigarette users, 12.7 cigarettes per day among former e-cigarette users and 19.9 cigarettes per day among never e-cigarette users (cigarettes per day not reported in the table).

**Table 4:** Tobacco and nicotine product use across three categories of cigarette smoking status (current, former, and never smoker): Population Assessment of Health and Tobacco (PATH) Study, 2013–2014.

Tobacco product	Cancer survivor (n = 1,527)			No history of cancer (n = 30,717)		
Cigarette smoking status	Current (n = 507)	Former (n = 416)	Never (n = 604)	Current (n = 10,872)	Former (n = 4,491)	Never (n = 15,354)
	Weighted % (95% CI)	Weighted % (95% CI)	Weighted % (95% CI)	Weighted % (95% CI)	Weighted % (95% CI)	Weighted % (95% CI)
**Cigarettes**						
Any cigarette smoker	100.0 (100.0, 100.0)	-	-	100.0 (100.0, 100.0)	-	-
Exclusive smoker	58.8 (53.3, 64.0)	-	-	54.2 (53.1, 55.4)	-	-
**E-cigarettes**						
Current user	22.0 (18.0, 26.5)	1.7 (1.1, 2.8)	0.9 (0.5, 1.4)	21.2 (20.4, 22.1)	4.2 (3.7, 4.8)	1.5 (1.4, 1.6)
Former user	2.6 (1.5, 4.6)	0.5 (0.3, 1.1)	0.3 (0.1, 0.7)	3.5 (3.1, 3.9)	1.5 (1.3, 1.7)	0.2 (0.2, 0.3)
Never user	75.4 (70.8, 79.5)	97.7 (96.6, 98.5)	98.8 (98.2, 99.2)	75.3 (74.4, 76.2)	94.3 (93.7, 94.8)	98.3 (98.2, 98.5)
**Any cigars**						
Current smoker	15.0 (11.5, 19.3)	4.0 (2.9, 5.4)	1.8 (1.3, 2.6)	20.6 (19.8, 21.6)	5.6 (5.1, 6.2)	4.3 (4.0, 4.6)
Former smoker	5.4 (3.4, 8.3)	8.3 (5.7, 12.0)	1.5 (0.8, 2.9)	5.6 (5.1, 6.1)	8.0 (7.0, 9.3)	1.2 (1.0, 1.4)
Never smoker	79.7 (74.9, 83.7)	87.7 (84.2, 90.5)	96.7 (95.2, 97.7)	73.8 (72.8, 74.8)	86.3 (85.0, 87.6)	94.5 (94.2, 94.8)
**Smokeless tobacco and snus**						
Current user	4.7 (3.3, 6.6)	1.0 (0.6, 1.7)	1.2 (0.8, 1.8)	7.0 (6.5, 7.7)	4.4 (3.9, 5.0)	2.0 (1.9, 2.3)
Former user	4.8 (3.2, 7.3)	3.7 (2.3, 5.7)	1.2 (0.5, 2.6)	5.7 (5.2, 6.2)	7.0 (6.0, 8.1)	1.5 (1.2, 1.8)
Never user	90.5 (87.5, 92.8)	95.3 (93.2, 96.8)	97.6 (96.3, 98.4)	87.3 (86.5, 88.1)	88.6 (87.4, 89.7)	96.5 (96.1, 96.8)
**Hookah**						
Current smoker	4.0 (2.5, 6.4)	0.4 (0.1, 1.1)	0.6 (0.3, 1.1)	10.0 (9.3, 10.8)	2.0 (1.7, 2.3)	3.3 (3.1, 3.6)
Former smoker	NA	0.3 (0.1, 0.9)	0.1 (0.0, 0.4)	1.5 (1.2, 1.7)	1.1 (0.9, 1.4)	0.5 (0.4, 0.6)
Never smoker	96.0 (93.7, 97.5)	99.3 (98.5, 99.7)	99.3 (98.8, 99.6)	88.5 (87.7, 89.3)	96.9 (96.4, 97.3)	96.2 (95.9, 96.4)
**Pipe tobacco**						
Current smoker	2.0 (1.0, 3.7)	0.9 (0.5, 1.6)	0.6 (0.3, 1.1)	3.1 (2.7, 3.6)	0.9 (0.7, 1.1)	0.6 (0.5, 0.7)
Former smoker	5.3 (3.4, 8.2)	12.1 (8.6, 16.9)	3.6 (2.3, 5.6)	2.5 (2.2, 2.8)	6.7 (5.7, 7.9)	0.9 (0.7, 1.2)
Never smoker	92.7 (89.9, 94.9)	87.0 (82.2, 90.7)	95.8 (93.8, 97.2)	94.4 (93.9, 94.9)	92.4 (91.2, 93.4)	98.5 (98.2, 98.7)

Abbreviation: NA, not available (suppressed)

Among non-cancer respondents who were current cigarette smokers (n = 10,872), 54.2% were exclusive cigarette smokers, whereas 21.2% were also current users of e-cigarettes, 20.6% were also current smokers of any cigars, 10.0% were also current hookah smokers, 7.0% were also current users of smokeless tobacco or snus, and 3.1% were also current smokers of pipe tobacco. On average, non-cancer respondents who were exclusive cigarette smokers reported smoking 15.7 cigarettes per day, compared with 16.8 cigarettes per day among current e-cigarette users, 16.7 cigarettes per day among former e-cigarette users and 17.2 cigarettes per day among never e-cigarette users.

**Fig 2:**
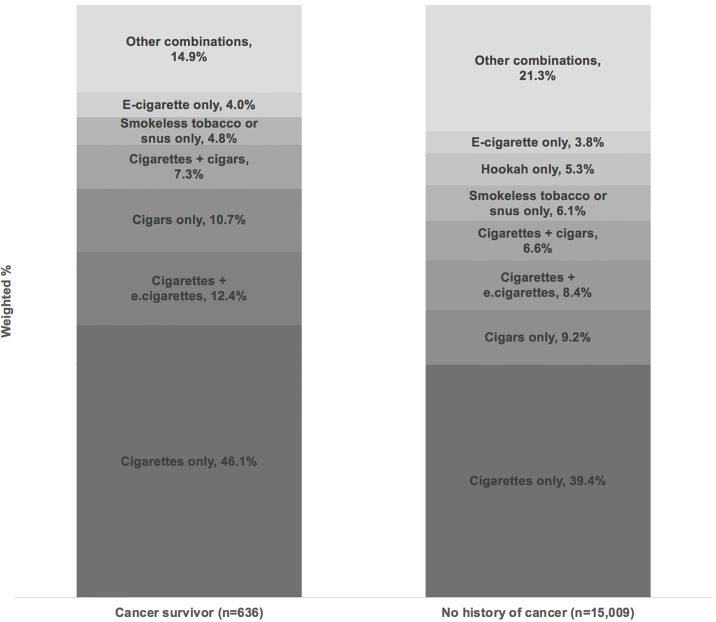
Tobacco/Nicotine product use among current users of any tobacco/nicotine products, by cancer status. Note: 44 cancer survivors and 766 adults with no history of cancer were excluded due to missing values for any tobacco or nicotine product use.

Fig 2 presents the most common combinations of tobacco and nicotine products used among current tobacco/nicotine product users, stratified by cancer survivors and non-cancer respondents. Among cancer survivors who reported current use of any tobacco or nicotine products (n = 636), the most common combinations were cigarettes only (46.1%), followed by cigarettes and e-cigarettes (12.4%). Among non-cancer respondents who reported current use of any tobacco or nicotine products (n = 15,009), the most common combinations were cigarettes only (39.4%), followed by cigars only (9.2%), and cigarettes and e-cigarettes (8.4%).

### Reasons for non-cigarette product use

The reasons for non-cigarette product use among cancer survivors are described in Table 5. Among e-cigarette users, the most commonly endorsed reason was “they might be less harmful to people around me than cigarettes” (85.0%). Among users of other products, a commonly endorsed reason was “they come in flavors I like”–which was endorsed by 97.8% among cigar smokers, 100.0% among smokeless tobacco/snus users, 80.9% among hookah smokers, and 66.4% among pipe smokers.

**Table 5:** Reasons for non-cigarette product use among cancer survivors: Population Assessment of Health and Tobacco (PATH) Study, 2013–2014.

Reasons for use among cancer survivors	Weighted % (95% CI)				
I use [product] because …	E-cigarettes (n = 189)	Any Cigars (n = 240)	Smokeless/snus (n = 120)	Hookah (n = 48)	Pipe tobacco (n = 139)
… they might be less harmful to me than cigarettes	82.1 (75.4, 87.4)	82.8 (70.3, 90.7)	87.2 (71.5, 94.9)	57.7 (42.9, 71.3)	44.1 (25.9, 64.1)
… they might be less harmful to people around me than cigarettes	85.0 (77.6, 90.2)	NA	89.9 (76.4, 96.0)	NA	NA
… using them helps people to quit smoking cigarettes	78.4 (72.6, 83.2)	65.7 (47.9, 80.0)	81.8 (61.2, 92.8)	17.5 (8.2, 33.7)	33.4 (16.7, 55.7)
… I can use them at times when or in places where smoking cigarettes isn’t allowed	82.1 (72.3, 89.0)	49.5 (31.0, 68.0)	94.8 (84.1, 98.4)	29.0 (17.4, 44.0)	17.2 (6.5, 38.5)
… they are more acceptable to non-tobacco users	71.1 (64.1, 77.1)	NA	87.5 (70.0, 95.5)	NA	NA
… they don’t smell	74.1 (63.3, 82.7)	NA	86.6 (70.5, 94.6)	NA	NA
… they come in flavors I like	56.9 (48.4, 65.0)	97.8 (92.4, 99.4)	100.0 (100.0, 100.0)	80.9 (65.8, 90.4)	66.4 (48.1, 80.8)
… they are affordable	53.6 (45.6, 61.3)	94.3 (85.8, 97.9)	78.8 (59.0, 90.6)	40.4 (25.3, 57.5)	49.6 (31.5, 67.8)
… using them feels like smoking a regular cigarette	55.9 (48.4, 63.2)	85.4 (70.6, 93.4)	NA	NA	NA
… I like socializing while using them	38.0 (29.9, 46.9)	95.8 (89.1, 98.5)	NA	82.8 (59.8, 94.0)	44.1 (26.4, 63.5)
… people in the media or other public figures use them	20.8 (14.6, 28.6)	60.3 (43.4, 75.1)	48.3 (20.5, 77.2)	21.0 (9.8, 39.3)	19.4 (7.9, 40.2)
… people who are important to me use them	20.7 (15.0, 27.8)	63.6 (44.6, 79.1)	69.3 (45.2, 86.0)	28.6 (17.2, 43.6)	10.9 (3.6, 28.2)
… the advertising for them appeals to me	17.5 (12.4, 24.0)	59.4 (40.2, 76.2)	48.0 (22.0, 75.1)	31.7 (18.0, 49.5)	18.4 (7.5, 38.7)

Abbreviation: NA, not asked

Noncigarette tobacco users and former users who quit in the past 12 months were asked to indicate (yes/no) whether particular reasons applied to their use of each specific product.

Questions regarding reasons for use were asked separately for past 30-day use of traditional cigar, cigarillo, and filtered cigar. Any respondents reporting past 30-day use of 2 or more types of cigars were asked to report on reasons for use for each type of cigar separately. Responses were aggregated so that if the reason was endorsed for any of the types of cigars, it was counted overall as a positive response.

### Correlates of any tobacco or nicotine product use

Results from the multivariable logistic regression modeling of current tobacco/nicotine product use stratified by cancer status are reported in Table 6. The factors associated with any current tobacco/nicotine use were similar in cancer survivors and non-cancer respondents. Compared with young adults (18–44 years), adults 45–64 and those ≥65 years old were less likely to be current tobacco/nicotine users in both cancer survivors and in non-cancer respondents. Males were more likely than females to be current tobacco users among both cancer survivors (AOR = 1.79, 95% CI = 1.39, 2.30) and non-cancer respondents (AOR = 2.10, 95% CI = 1.99, 2.22). Whereas black non-cancer respondents were less likely to be current tobacco/nicotine users than whites (AOR = 0.90, 95% CI = 0.82, 0.99), there were no significant differences in current tobacco/nicotine use between black and white cancer survivors (AOR = 0.88, 95% CI = 0.58, 1.31). Compared with non-Hispanic whites, respondents of other racial/ethnic groups were less likely to be current tobacco/nicotine users in non-cancer respondents (AOR = 0.78, 95% CI = 0.69, 0.88).

**Table 6:** Multivariable logistic regression models of any (≥1 products vs. none) and dual/poly (≥2 products vs. 1 product) tobacco/nicotine product use, by cancer status: Population Assessment of Health and Tobacco (PATH) Study, 2013–2014.

	Cancer survivor (n = 1,527)				No history of cancer (n = 30,717)			
	Any use		Dual/poly use		Any use		Dual/poly use	
Characteristics	OR (95% CI)	*P*	OR (95% CI)	*P*	OR (95% CI)	*P*	OR (95% CI)	*P*
**Age, in years**								
18–44	Reference		Reference		Reference		Reference	
45–64	0.44 (0.30, 0.64)	<0.001	0.56 (0.35, 0.89)	0.015	0.67 (0.63, 0.72)	<0.001	0.53 (0.49, 0.59)	<0.001
≥65	0.12 (0.08, 0.17)	<0.001	0.26 (0.14, 0.49)	<0.001	0.23 (0.21, 0.25)	<0.001	0.30 (0.24, 0.37)	<0.001
**Sex**								
Female	Reference		Reference		Reference		Reference	
Male	1.79 (1.39, 2.30)	<0.001	1.19 (0.80, 1.78)	0.395	2.10 (1.99, 2.22)	<0.001	1.59 (1.47, 1.73)	<0.001
**Race**								
White, non-Hispanic	Reference		Reference		Reference		Reference	
Black, non-Hispanic	0.88 (0.58, 1.33)	0.551	0.84 (0.42, 1.71)	0.634	0.90 (0.82, 0.99)	0.023	0.94 (0.83, 1.06)	0.312
Other	0.65 (0.32, 1.31)	0.225	2.28 (0.99, 5.27)	0.054	0.78 (0.69, 0.88)	<0.001	1.11 (0.98, 1.26)	0.092
**Education**								
Not high school graduate	Reference		Reference		Reference		Reference	
GED or high school graduate	0.56 (0.36, 0.88)	0.012	0.99 (0.58, 1.67)	0.959	1.02 (0.92, 1.14)	0.689	1.04 (0.92, 1.18)	0.479
Some college or associate degree	0.55 (0.35, 0.87)	0.010	1.25 (0.69, 2.28)	0.453	0.92 (0.82, 1.02)	0.118	1.22 (1.07, 1.38)	0.003
Bachelor’s degree or higher	0.23 (0.13, 0.40)	<0.001	1.27 (0.57, 2.83)	0.551	0.43 (0.38, 0.49)	<0.001	0.98 (0.84, 1.14)	0.788
**Household income**								
<$25,000	Reference		Reference		Reference		Reference	
$25,000 to $49,999	0.80 (0.55, 1.15)	0.221	0.65 (0.38, 1.11)	0.113	0.89 (0.82, 0.96)	0.004	0.89 (0.81, 0.98)	0.020
$50,000 to $99,999	0.58 (0.41, 0.81)	0.002	1.31 (0.77, 2.25)	0.317	0.67 (0.62, 0.72)	<0.001	0.83 (0.75, 0.92)	0.001
$100,000 or more	0.53 (0.36, 0.78)	0.002	0.34 (0.16, 0.75)	0.008	0.57 (0.52, 0.63)	<0.001	0.68 (0.60, 0.77)	<0.001
**U.S. Census region**								
Northeast	Reference		Reference		Reference		Reference	
Midwest	1.01 (0.72, 1.42)	0.952	1.44 (0.69, 3.01)	0.333	1.15 (1.05, 1.26)	0.004	1.17 (1.05, 1.30)	0.006
South	0.99 (0.66, 1.49)	0.973	1.88 (0.94, 3.79)	0.074	1.02 (0.94, 1.11)	0.557	1.15 (1.04, 1.27)	0.006
West	1.10 (0.77, 1.59)	0.596	1.39 (0.58, 3.34)	0.461	0.75 (0.67, 0.85)	<0.001	1.20 (1.07, 1.34)	0.002
**Tobacco-related cancer**								
No	Reference		Reference		-	-	-	-
Yes	1.58 (1.18, 2.11)	0.002	1.19 (0.76, 1.88)	0.440	-	-	-	-

Adults with higher than a high school education were less likely than those without a high school degree to be current tobacco/nicotine users regardless of cancer history. For example, adults with a bachelor’s degree or higher were less likely than the referent education level to be current tobacco/nicotine users in both cancer survivors (AOR = 0.23, 95% CI = 0.13, 0.40) and in non-cancer respondents (AOR = 0.43, 95% CI = 0.38, 0.49). Additionally, adults with higher income were less likely to be current tobacco/nicotine users compared to adults with a lower annual household income. Among cancer survivors, those with a tobacco-related cancer diagnosis were more likely to be current tobacco/nicotine users (AOR = 1.58, 95% CI = 1.18, 2.11) than those diagnosed with a non-tobacco-related cancer.

### Correlates of dual and poly tobacco/nicotine use

In general, among those who reported current use of any tobacco or nicotine products, the correlates of dual and poly tobacco/nicotine use (vs. mono use) were similar to those variables associated with any tobacco or nicotine product use, with some exceptions (Table 6). First, there were no significant differences by sex in dual use among cancer survivors. However, among cancer survivors, education level was not significantly associated with dual or poly use and only membership in the highest annual household income level ($100,000 or more) was associated with lower odds of dual tobacco/nicotine use (AOR = 0.34, 95% CI = 0.16, 0.75). Meanwhile, having a tobacco-related cancer diagnosis was not significantly associated with the likelihood of being a dual or poly tobacco/nicotine user.

## Discussion

This cross-sectional analysis of tobacco-use behaviors among adult cancer survivors compared with non-cancer respondents provides an update on the prevalence of cigarette smoking and e-cigarette use, in addition to benchmark estimates of current and former use of other tobacco products in this population. Among adult cancer survivors participating in the PATH Study, approximately 17% are current tobacco users, including 13% who are current cigarette smokers, 5% who are current e-cigarette users, and 2% who are current cigar smokers. Many correlates of tobacco use in cancer survivors are consistent with those in the general population of U.S. adults: younger age, male gender, and lower household income.[16] In addition, cancer survivors diagnosed with tobacco-related cancers were more likely to be current smokers.

The estimated prevalence of current cigarette smoking among adult cancer survivors is consistent with estimates published by the National Cancer Institute[17]—12.8% in 2014 (data source: National Health Interview Survey [NHIS]). Despite overall and cancer-specific declines in cigarette smoking rates according to the NHIS, cigarettes remain the most commonly used tobacco product among cancer survivors across all age groups. Meanwhile, our analysis highlights differences in patterns by age group, with higher prevalence of e-cigarette and non-cigarette tobacco product use in younger cancer survivors as compared with older survivors. While specific evidence on the health effects of non-cigarette products in cancer survivors remains scarce, both combustible and non-combustible products have been associated with significant health risks in the general population.[1]

Although many correlates of tobacco use were consistent across cancer survivors and non-cancer participants, there were notable differences. Racial/ethnic and geographical associations of tobacco product use appeared to be more pronounced for non-cancer participants compared with cancer survivors, suggesting that cancer survivors nationwide exhibited less disparate tobacco use patterns than adults who did have a history of cancer. However, more research is needed to examine the racial/ethnic and geographic patterns in tobacco use among cancer survivors.

A previous analysis of the PATH Study data had focused on investigating e-cigarette use in cancer survivors who were cigarette smokers.[12] Similar to that study, we observed no differences in the prevalence of e-cigarettes among cancer survivors when compared to non-cancer participants. The National Academies of Science, Engineering, and Medicine (NASEM) recently detailed concerns about the use of e-cigarettes[18] and concern for the use of e-cigarettes by cancer patients has been raised by the International Association for the Study of Lung Cancer (IASLC).[19] However, while there are concerns about e-cigarette use after a cancer diagnosis, the adverse effects of continued smoking are unquestionable[1] and the use patterns in older adults strongly favors continued cigarette smoking over other tobacco products.

Our study took a broader approach than Symes et al.[12] by characterizing the prevalence of cigarettes, e-cigarettes, and four other categories of tobacco products in cancer survivors. The data demonstrate that 13% of cancer survivors report current cigarette smoking and nearly 17% use at least one tobacco or nicotine product. These rates appear to be lower than the 18–20% rates from the NHIS and HINTS data[3,4] which suggest a decreasing trend for tobacco use in cancer survivors, while highlighting the rise in alternative tobacco product use, especially among younger cancer survivors. Among cancer survivors who were current tobacco/nicotine users, nearly one-third were using two or more products, a rate that is similar to that among non-cancer respondents. This finding was consistent with the results of the Fahey et al. study which reported on a regional sample of cancer patients.[9] Further, our study identified the continued use of alternative tobacco products among cancer survivors who are former or never smokers, underscoring the need for interventions that extend beyond addressing cigarette smoking in this population.

As described in the Symes et al. study,[12] a majority of cancer survivors reported using e-cigarettes for perceived health-related reasons. Our study extended this finding to smokeless tobacco and snus users, as well as cigar smokers. Among both groups, a majority endorsed the statements related to harm reduction and cigarette smoking cessation. Additionally, a majority of hookah users shared the perception of harm reduction.

Although we found the prevalence of cigarette smoking and any product use to be lower among cancer survivors compared with non-cancer participants in the PATH Study, almost one in five cancer survivors reported current use of a tobacco or nicotine product. Continued tobacco use by cancer patients not only increases the risk for adverse cancer treatment outcomes,[1] but also significantly increases the cost of subsequent cancer treatment.[2] This study underscores the need for the integration of tobacco dependence treatment strategies in cancer care settings.[20–22] It also provides clinicians in cancer care settings with population-based benchmark estimates of cigarette and non-cigarette tobacco product use among cancer survivors. The American Society of Clinical Oncology (ASCO) and the National Comprehensive Cancer Network (NCCN) both recommend universal assessment and documentation of tobacco use for cancer patients in all clinical settings, as well as the provision of tobacco use treatment.[23,24] A large survey of oncologists by the IASLC demonstrates that while the majority of oncologists ask about tobacco and advise patients to quit, few assist patients with quitting.[25] A lack of time, training, and resources are predictive barriers to providing cessation support by oncologists.[26] Long term follow-up data from the Cancer Prevention Study II between 1992–2009 demonstrated a higher two-year quit rate in cancer survivors (31%) as compared with the general population (20%).[27] However, these results were outside of the context of a structured smoking cessation intervention. Recognizing the deficits in providing cessation support, the NCI recently funded the development of tobacco treatment programs at 42 NCI-Designated Cancer Centers.[28] This effort is expected to implement and sustain evidence-based smoking cessation on a broad scale for cancer centers, but it will take several years to realize results.

There are several limitations to this study. First, the PATH Study relies on self-reported cancer diagnosis, which typically underestimates cancer prevalence.[29] Tobacco and e-cigarette use is also based on self-reports, and data have shown that approximately 30% of cancer patients who smoke may misrepresent tobacco use.[30–32] Data are likely to underestimate tobacco use in this cohort, but the same risks are true for prior studies that have evaluated smoking in cancer cohorts.[30–32] This cross-sectional study design cannot support causal inferences and does not allow for the examination of the extent and timing of changes in tobacco use status among cancer survivors. This study is further limited by the inability to examine tobacco use in relation to time since diagnosis for cancer survivors. Furthermore, given the fast-changing landscape of e-cigarettes and emerging alternative tobacco products use, patterns from 2013–2014 may not reflect the latest experiences of these products among the US population. Despite these limitations, the current study provides new knowledge regarding the prevalence of tobacco and nicotine product use among adult cancer survivors in the U.S. from the well-designed and broadly implemented PATH Study.

## Conclusions

Findings from the current study demonstrate dynamic use patterns for cigarettes, e-cigarettes, and other tobacco products among cancer survivors compared with the general population. As data from the PATH Study mature, future analyses can evaluate changes in patterns of use and begin to better define opportunities for assessment of health risk and intervention.
